# Identification of ISZ-sTRAIL Protein as a Potent Anticancer Agent for EML4-ALK-Positive Non-Small-Cell Lung Cancer

**DOI:** 10.3390/molecules31111870

**Published:** 2026-05-29

**Authors:** Junfeng Hu, Junhui Guo, Tian Qin, Xiuping Mao, Zi Liu, Liang Ma

**Affiliations:** Department of Chemical Biology and Pharmaceutical Engineering, School of Chemistry and Chemical Engineering, Anhui University of Technology, Ma’anshan 243002, China; 18755108016@163.com (J.H.); 18061860258@163.com (J.G.); 13083109359@163.com (T.Q.); 18348473707@163.com (X.M.)

**Keywords:** ISZ-sTRAIL protein, EML4-ALK-positive non-small-cell lung cancer, apoptosis, caspase activation

## Abstract

EML4-ALK-positive lung cancer represents an important molecular subtype of non-small-cell lung cancer (NSCLC) that initially responds to ALK inhibitors but invariably develops resistance, highlighting the need for novel targeted therapeutic strategies. Death receptors DR4 and DR5 are frequently upregulated in malignancies and can selectively induce tumor cell apoptosis upon binding TRAIL. ISZ-sTRAIL, a trimer-stabilized soluble TRAIL fusion protein, exhibits potent antitumor effects via DR4/DR5 signaling activation. However, the expression status of DR4/DR5 in EML4-ALK-positive NSCLC cells and the therapeutic potential of targeting this pathway remain poorly defined. In this study, we evaluated DR4/DR5 protein expression in EML4-ALK-positive NSCLC cells and investigated the antitumor effect of ISZ-sTRAIL produced in an *Escherichia coli* expression system. Our results showed that DR4 and DR5 were abundantly expressed in EML4-ALK-positive NCI-H2228 and NCI-H3122 cells compared with normal human bronchial epithelial 16HBE cells. Furthermore, ISZ-sTRAIL significantly suppressed the proliferation of NCI-H2228 and NCI-H3122 cells, with IC_50_ values of 4.51 ± 0.22 nM and 14.98 ± 3.34 nM, respectively, while showing low cytotoxicity toward normal 16HBE cells (IC_50_ > 1 μM). Moreover, ISZ-sTRAIL induced caspase-dependent apoptosis in both cell lines via activation of extrinsic and intrinsic pathway, and these effects were markedly abrogated by the pan-caspase inhibitor Z-VAD. These findings identify DR4/DR5 as a potential therapeutic target and provide preclinical evidence for the development of TRAIL-based strategies in the treatment of EML4-ALK-positive NSCLC.

## 1. Introduction

Anaplastic lymphoma kinase (ALK) rearrangement, identified in approximately 3–7% of non-small-cell lung cancer (NSCLC) cases, represents the second most prevalent oncogenic driver in this disease after EGFR mutations [1]. These genomic rearrangements give rise to oncogenic fusion proteins, thereby establishing ALK as a pivotal therapeutic target for NSCLC. Such oncoproteins are typically formed by the fusion of the 3′ kinase-encoding domain of ALK with the 5′ region of various partner genes. Among these partner genes, echinoderm microtubule-associated protein-like 4-ALK (EML4-ALK) represents the earliest identified and most prevalent variant, accounting for up to 85% of all ALK-rearranged NSCLC cases [2,3]. The oncogenic potential of ALK fusion proteins arises predominantly from the constitutive activation of their intrinsic receptor tyrosine kinase (RTK) function, which promotes tumor initiation and progression through the sustained activation of downstream pro-proliferative signaling cascades, including the MAPK, JAK/STAT, and PI3K/AKT pathways [4]. Consequently, ALK tyrosine kinase inhibitors have been established as the standard first-line therapy for this molecular subtype of NSCLC, with three generations of inhibitors sequentially introduced into clinical practice and fourth-generation ALK inhibitors currently under clinical development [5]. Despite remarkable initial responses, nearly all patients eventually develop acquired resistance to ALK-targeted therapies. Analogous to the developmental paradigm employed for other kinase-targeted drugs including EGFR inhibitors, current strategies to combat resistance largely center on the development of next-generation inhibitors with enhanced potency and selectivity towards ALK activity [6]. Nonetheless, the rapid onset of secondary resistance remains a fundamental unresolved challenge, underscoring the critical need to explore innovative therapeutic strategies based on novel molecular targets and alternative mechanisms of action.

Protein engineering technologies allow for substantial optimization of the stability, targeting specificity, and biological functions of biomacromolecules via rational structural modification, and they have become an important strategy for tumor therapy [7]. Tumor necrosis factor-related apoptosis-inducing ligand (TRAIL), a member of the TNF superfamily, can selectively bind to death receptors DR4/DR5, thereby activating the apoptotic pathway to induce cell death [8,9]. Studies have shown that DR4/DR5 are highly expressed in various cancers but at low levels in normal cells. Therefore, TRAIL exerts specific cytotoxicity against a variety of tumor cells with low toxicity to normal cells [10]. In addition, the soluble form of TRAIL (sTRAIL) also has antitumor activity. Owing to its relatively small molecular weight and the ability to be efficiently expressed at high yields in *Escherichia coli*, it holds great promise for further applications and development [11]. However, sTRAIL protein is limited by a short half-life and competitive inhibition by decoy receptors (e.g., DcR1) within the tumor microenvironment, which significantly impairs its antitumor activity. To overcome these defects, researchers have constructed engineered sTRAIL fusion proteins [12]. For example, conjugating trimerization domains such as isoleucine zipper (ISZ) to the N-terminus of sTRAIL can significantly enhance its trimer stability and biological activity [13,14,15,16]. Similarly, fusion of the iRGD peptide to sTRAIL yields sTRAIL-iRGD with improved tumor-penetrating ability [17]. Moreover, bifunctional proteins such as SAC-TRAIL have been developed to prolong the circulating half-life and augment antitumor efficacy [18]. Notably, aponermin, a circularly permuted TRAIL (CPT) with enhanced stability and extended half-life, was initially approved in China in November 2023 as a combination therapy with thalidomide and dexamethasone for relapsed or refractory multiple myeloma [19]. Nevertheless, whether sTRAIL-based proteins can be developed as a potential anticancer therapeutic strategy against EML4-ALK-positiveNSCLC remains unclear.

In the present study, we examined the expression levels of death receptors DR4 and DR5 in EML4-ALK-positive NSCLC cells and found that both receptors were significantly upregulated compared with normal lung epithelial cells. We constructed prokaryotic expression vectors for ISZ-sTRAIL and sTRAIL and obtained the purified recombinant proteins from *Escherichia coli*. Using these proteins as candidate therapeutic agents, we further evaluated their antitumor activity and underlying mechanism in EML4-ALK-positive NSCLC cells, aiming to develop novel therapeutic strategies for this subtype of lung cancer.

## 2. Results

### 2.1. Construction and Expression of sTRAIL and ISZ-sTRAIL Protein

**Figure 1:**
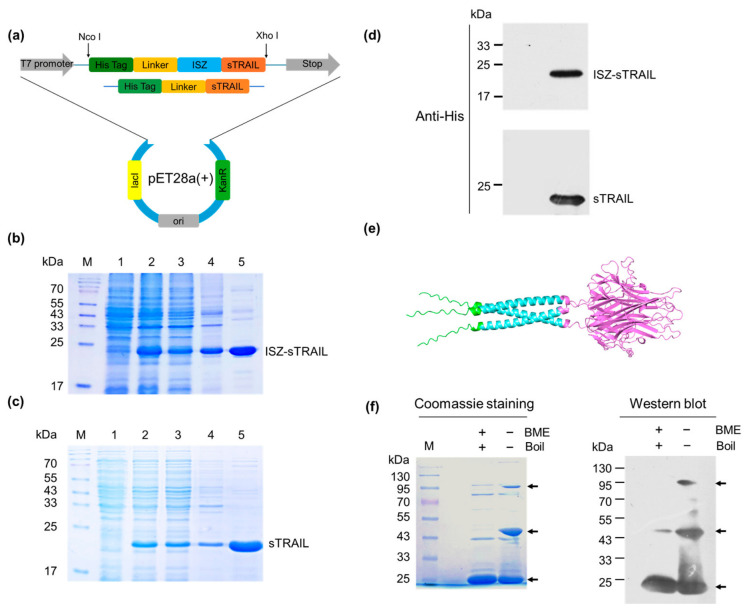
Expression and purification of recombinant ISZ-sTRAIL and sTRAIL fusion protein. (**a**) Schematic diagram of the ISZ-sTRAIL and sTRAIL expression vectors; (**b**,**c**) SDS-PAGE analysis of ISZ-sTRAIL (**b**) and sTRAIL (**c**) expression and purification: M, protein marker; Lane 1, total cell lysate without isopropyl β-D-1-thiogalactopyranoside (IPTG) induction; Lane 2, total cell lysate with IPTG induction; Lane 3, supernatant of induced cell lysate; Lane 4, pellet of induced cell lysate; Lane 5, purified protein eluted with 500 mM imidazole; (**d**) Western blot analysis of ISZ-sTRAIL and sTRAIL using an anti-His tag antibody; (**e**) three-dimensional structure of ISZ-sTRAIL trimer predicted by AlphaFold 3 and visualized using PyMOL. The blue and purple regions indicate ISZ and sTRAIL, respectively; (**f**) identification of polymeric ISZ-sTRAIL fusion protein. The ISZ-sTRAIL protein was mixed with the sample buffer with or without β-mercaptoethanol (BME), separated on 10% SDS−PAGE gels, and detected by Coomassie Blue staining (left panel) and Western blot (right panel) using an anti-His antibody. Arrows from bottom to top indicate the monomeric, as well as the putative dimeric and trimeric forms of ISZ-sTRAIL, respectively.

The sTRAIL and ISZ-sTRAIL expression vectors (Figure 1a) were successfully constructed and transformed into *Escherichia coli BL21(DE3)* competent cells. The target protein was purified by nickel-column affinity chromatography and analyzed by SDS-PAGE. As displayed in Figure 1b, the ISZ-sTRAIL fusion protein was clearly detected in the supernatant of the cell lysate (Lane 3, Figure 1b), indicating that the fusion protein was expressed in a soluble form in the *E. coli* expression system. Following nickel-column purification, the ISZ-sTRAIL fusion protein was eluted with imidazole (Lane 5, Figure 1b), and its molecular weight was consistent with the theoretical value of approximately 24.8 kDa. Native sTRAIL (the predicted molecular weight is approximately 21.3 kDa) was also successfully purified (Figure 1c). To further verify the correct expression of the obtained proteins, Western blot analysis was performed. As shown in Figure 1d, both ISZ-sTRAIL and sTRAIL proteins were specifically recognized by the anti-His tag antibody, confirming that both the sTRAIL and ISZ-sTRAIL vectors were correctly expressed. In addition, the three-dimensional structure of ISZ-sTRAIL (Figure 1e) was predicted using the AlphaFold 3 server [20] and visualized by PyMOL 3.0.3 software. ISZ-sTRAIL is composed of an α-helical region (ISZ) and multiple β-sheet regions (sTRAIL), and the trimeric assembly of the fusion protein is clearly illustrated in the structure. Furthermore, as revealed by nonreducing Coomassie Blue staining and Western blot (Figure 1f), the ISZ-sTRAIL fusion protein exhibited a polymerized structure in its native state, likely including dimers or trimers. This polymeric pattern is similar to that previously reported for sTRAIL multimers [13,21].

### 2.2. In Vitro Cytotoxicity of ISZ-sTRAIL Protein

**Figure 2:**
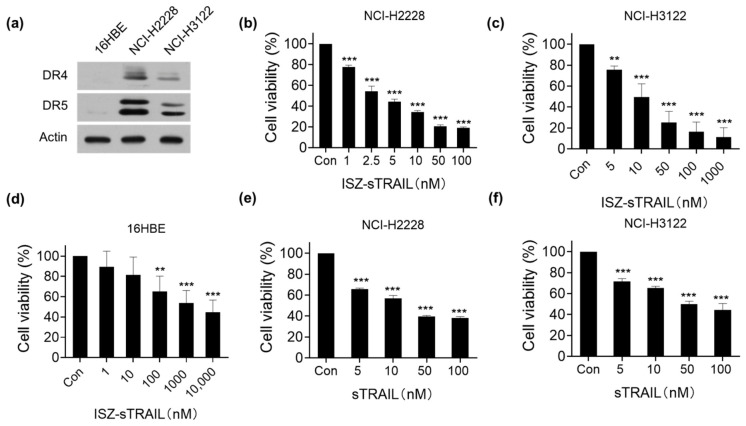
The cytotoxic effect of ISZ-sTRAIL and sTRAIL protein against EML4-ALK-positive NSCLC cells. (**a**) Expression levels of TRAIL receptors DR4 and DR5 in NCI-H3122, NCI-H2228, and 16HBE cells; (**b**–**d**) the cytotoxic effect of ISZ-sTRAIL on EML4-ALK-positive NSCLC cells NCI-H2228 (**b**), NCI-H3122 (**c**), and the normal human bronchial epithelial 16HBE (**d**) cells; (**e**,**f**) the cytotoxic effect of sTRAIL on NCI-H2228 (**e**) and NCI-H3122 (**f**) cells. Data are presented as mean ± SD of at least three independent experiments. ** *p* < 0.01, *** *p* < 0.001 vs. control.

NCI-H3122 and NCI-H2228 are two commonly used cell lines representing ALK-rearranged NSCLC, each harboring distinct EML4-ALK fusion variants. NCI-H3122 cells carry EML4-ALK variant 1 (V1), in which exon 13 of EML4 (E13) is fused to exon 20 of ALK (A20), designated E13;A20, whereas NCI-H2228 cells harbor the E6a/b;A20 fusion variant [22]. Accordingly, these two cell lines were selected as models to investigate the anticancer activity of ISZ-sTRAIL protein. Firstly, the expression of the TRAIL receptors DR4 and DR5 in the three cell lines was detected. As shown in Figure 2a, both DR4 and DR5 were highly expressed in EML4-ALK-positive NSCLC cells, whereas their expression levels were low in normal human bronchial epithelial 16HBE cells. The cytotoxicity of ISZ-sTRAIL protein against NCI-H2228 and NCI-H3122 cells was then examined by MTT assay. As illustrated in Figure 2b,c, ISZ-sTRAIL inhibited the proliferation of these lung cancer cells in a dose-dependent manner. The corresponding IC_50_ values, summarized in Table 1, revealed that ISZ-sTRAIL exhibited potent inhibitory activity, with IC_50_ values of 4.51 ± 0.22 nM in NCI-H2228 cells and 14.98 ± 3.34 nM in NCI-H3122 cells. Furthermore, the effects of ISZ-sTRAIL on normal 16HBE cells were also evaluated. The results demonstrated that its cytotoxicity toward 16HBE cells was markedly lower than that toward EML4-ALK-positive NSCLC cells (Figure 2d and Table 1), with an IC_50_ value above 1 μM. To determine whether the polymeric design of ISZ-sTRAIL enhances its bioactivity, we compared its cytotoxic effect with that of native sTRAIL. Cell viability assays showed that sTRAIL exhibited weaker inhibitory activity against NCI-H2228 and NCI-H3122 cells compared with ISZ-sTRAIL (Figure 2e,f and Table 1), indicating that the ISZ modification effectively promotes protein multimerization and enhances antitumor activity. These findings indicate that ISZ-sTRAIL exerts significant cytotoxicity against EML4-ALK-positive NSCLC cells while displaying low toxicity to normal cells.

### 2.3. ISZ-sTRAIL Inhibits the Proliferation of EML4-ALK-Positive NSCLC Cells

**Figure 3:**
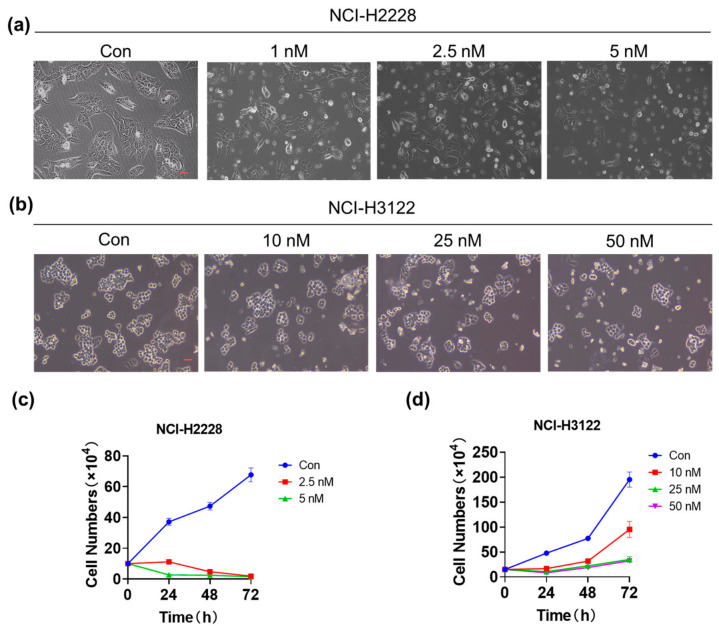
ISZ-sTRAIL reduces the viability of EML4-ALK-positive NSCLC cells. (**a**,**b**) Morphological changes in NCI-H2228 (**a**) and NCI-H3122 (**b**) cells following ISZ-sTRAIL treatment. Scale bars, 50 μm; (**c**,**d**) viability of NCI-H2228 (**c**) and NCI-H3122 (**d**) cells detected by Trypan blue staining after treatment with ISZ-sTRAIL at different concentrations and time points. Data are presented as mean ± SD of three independent experiments.

The morphological results in Figure 3a,b show that ISZ-sTRAIL significantly altered cell morphology, characterized by cell rounding, decreased adherence, and a reduction in the number of NCI-H2228 and NCI-H3122 cells. Trypan blue staining analysis revealed that, compared with the untreated control group, the number of viable cells of both cell lines significantly decreased with the increase in ISZ-sTRAIL concentration and the extension of treatment time (see Figure 3c,d). The observed morphological changes coupled with reduced viable cell counts indicate that ISZ-sTRAIL exerts potent cytotoxic effects against NCI-H2228 and NCI-H3122 cells.

### 2.4. ISZ-sTRAIL Induces Apoptosis of EML4-ALK-Positive NSCLC Cells

**Figure 4:**
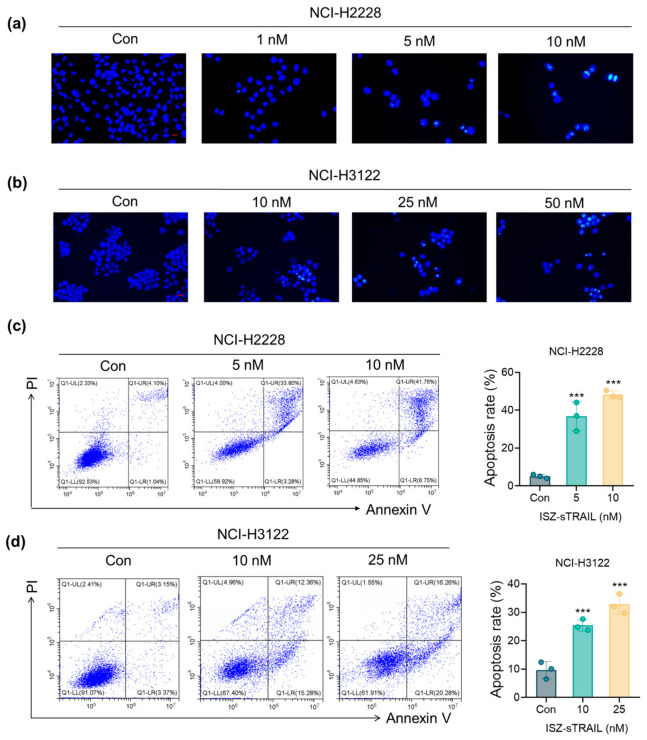
Induction of apoptosis in EML4-ALK-positive NSCLC cells by ISZ-sTRAIL. (**a**,**b**) Hoechst 33342 fluorescence staining showing nuclear changes in NCI-H2228 (**a**) and NCI-H3122 (**b**) cells following ISZ-sTRAIL treatment. Scale bars, 20 μm; (**c**,**d**) apoptotic effects of ISZ-sTRAIL on NCI-H2228 (**c**) and NCI-H3122 (**d**) cells assessed by flow cytometry. Data are presented as mean ± SD of three independent experiments. *** *p* < 0.001 vs. control.

TRAIL binds to its receptors DR4 and DR5 to induce cell apoptosis. We therefore further investigated whether the ISZ-sTRAIL protein could trigger apoptosis in EML4-ALK-positive NSCLC cells. Hoechst fluorescence staining results (Figure 4a,b) showed that after ISZ-sTRAIL treatment, obvious nuclear condensation was observed in both cell lines, and some cells exhibited nuclear fragmentation, which suggested that cell apoptosis occurred. Flow cytometry analysis (Figure 4c,d) further confirmed that treatment with ISZ-sTRAIL elevated the apoptotic cell proportion in NCI-H2228 and NCI-H3122 cells in a dose-dependent manner. In NCI-H3122 cells, ISZ-sTRAIL (10 and 25 nM) induced apoptosis rates of 26.05% and 33.45%, respectively, whereas in NCI-H2228 cells, treatment with 5 and 10 nM ISZ-sTRAIL resulted in apoptosis rates of 36.76% and 48.28%, respectively. Collectively, these results demonstrate that ISZ-sTRAIL effectively induces apoptosis in both EML4-ALK-positive NSCLC cell lines in a dose-dependent manner.

### 2.5. ISZ-sTRAIL Activates the Caspase Pathway

**Figure 5:**
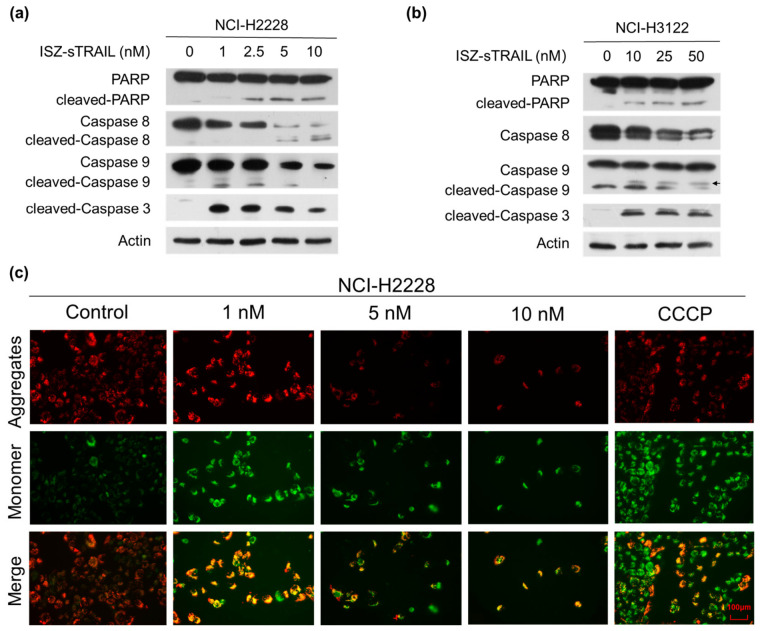
Activation of the caspase pathway by ISZ-sTRAIL in EML4-ALK-positive NSCLC cells. Western blot analysis was performed to detect the cleavage of caspase 8, caspase 9, caspase 3, and PARP in NCI-H2228 (**a**) and NCI-H3122 (**b**) cells after ISZ-sTRAIL treatment. Actin was used as an internal reference. The arrow indicates the cleaved-Caspase9 band; (**c**) MMP changes in NCI-H2228 cells treated with ISZ-sTRAIL for 24 h, assessed by JC-1 staining. Red fluorescence indicates normal MMP (JC-1 aggregates), while green fluorescence indicates MMP loss (JC-1 monomers). Scale bar, 100 μm.

Given that the caspase signaling cascade represents the canonical pathway mediating TRAIL-induced apoptosis, we further examined the activation of key components in this pathway. After treatment with ISZ-sTRAIL, marked cleavage or reduction of caspase-8, caspase-9, caspase-3, and PARP was detected in both NCI-H2228 and NCI-H3122 cells (Figure 5a,b). Moreover, using the JC-1 fluorescent probe, we observed via fluorescence microscopy that ISZ-sTRAIL treatment dose-dependently reduced the mitochondrial membrane potential (MMP) (Figure 5c). As caspase 8 and caspase 9 serve as key initiators of the extrinsic and intrinsic apoptotic pathways, respectively, and given the observed loss of MMP, these results indicate that ISZ-sTRAIL induces apoptosis in EML4-ALK-positive NSCLC cells through the activation of both extrinsic and intrinsic caspase cascades.

### 2.6. Caspase-Dependent Apoptosis Mediates the Anti-EML4-ALK-Positive NSCLC Activity of ISZ-sTRAIL

**Figure 6:**
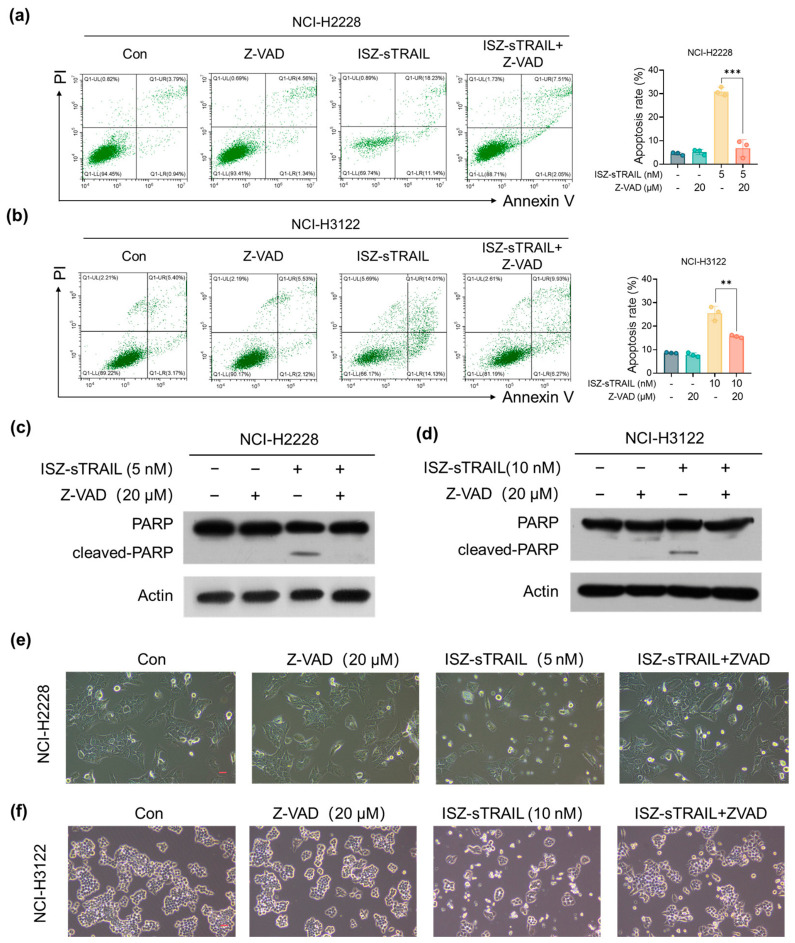
Caspase-dependent apoptosis is involved in the anti-EML4-ALK-positive NSCLC activity of ISZ-sTRAIL. (**a**,**b**) Flow cytometry analysis showing the effect of Z-VAD on ISZ-sTRAIL-induced apoptosis in NCI-H2228 (**a**) and NCI-H3122 (**b**) cells. ** *p* < 0.01, *** *p* < 0.001 (n = 3); (**c**,**d**) Western blot analysis of PARP expression in NCI-H2228 (**c**) and NCI-H3122 (**d**) cells treated with ISZ-sTRAIL, Z-VAD, or their combination; (**e**,**f**) morphological changes in NCI-H2228 (**e**) and NCI-H3122 (**f**) cells under different treatment conditions. Scale bars, 50 μm.

To further clarify the critical role of caspase pathway activation in the anti-EML4-ALK-positive NSCLC activity of ISZ-sTRAIL, the pan-caspase inhibitor Z-VAD-FAM (Z-VAD) was applied. We first detected whether Z-VAD affected ISZ-sTRAIL-induced apoptosis using flow cytometry. As shown in Figure 6a,b, Z-VAD partially reversed the apoptosis-inducing effect of ISZ-sTRAIL in both cell lines. Subsequently, Western blot analysis was performed to detect changes in the expression level of the apoptosis-related protein PARP. As illustrated in Figure 6c,d, treatment with ISZ-sTRAIL alone significantly induced PARP cleavage, whereas such cleavage was almost completely abrogated by pretreatment with Z-VAD. We then explored whether the morphological changes in cells treated with ISZ-sTRAIL could be reversed in the presence of Z-VAD. As shown in Figure 6e,f, the number of cells treated with ISZ-sTRAIL alone was significantly reduced, with cells rounding up and showing a tendency to detach. However, when ISZ-sTRAIL was applied to cells pretreated with Z-VAD, the morphology and number of cells were restored to a state similar to that of the control group. Collectively, these results demonstrate that ISZ-sTRAIL induces caspase-dependent apoptosis in EML4-ALK-positive NSCLC cells.

## 3. Discussion

The discovery of ALK rearrangements as a driver oncogene in 2007 marked a pivotal moment in targeted therapy for EML4-ALK-positive NSCLC. However, acquired resistance inevitably develops in almost all patients, representing a major clinical challenge. The mechanisms of resistance to ALK tyrosine kinase inhibitors are multifaceted, encompassing on-target mutations within the ALK domain and activation of the bypass signaling pathway [23]. While immunotherapy has brought new hope for NSCLC treatment, the immunosuppressive tumor microenvironment in ALK-positive NSCLC enables immune evasion, thereby contributing to therapeutic resistance [24,25]. These mechanisms collectively underscore the urgent need for alternative treatment strategies for EML4-ALK-positive NSCLC.

An effective strategy to generate trimeric TRAIL is to fuse it with domains capable of spontaneous trimerization, such as the leucine zipper (LZ) or its isoleucine zipper (ISZ) sequence, which is known to be a strong trimerization domain and relatively smaller in size than LZ. Previous studies have demonstrated that secretory expression of ISZ-sTRAIL in 293T cells produced secretable trimeric forms of TRAIL proteins, which exhibited higher apoptotic activity in HeLa cells [26]. Using the *P. pastoris* yeast expression system, LZ-TRAIL was successfully secreted and purified, and the highly stable trimeric TRAIL protein showed significantly enhanced anticancer activity [16]. In addition, fusion of the ISZ hexamerization motif to the N-terminus of TRAIL enabled multimerization of its trimeric form and exhibited higher cytotoxic activity compared with the native form [21]. Despite these advances, the efficacy of ISZ-sTRAIL in EML4-ALK-positive NSCLC cells has remained unexplored. In this study, we constructed a prokaryotic expression vector for the ISZ-sTRAIL fusion protein and successfully obtained the purified recombinant protein from *Escherichia coli*. Notably, ISZ-sTRAIL demonstrated potent cytotoxicity against EML4-ALK-positive NSCLC cells. Importantly, consistent with the trimerization-enhancing strategy described above, our data revealed that ISZ promotes sTRAIL multimerization, which likely underlies the enhanced anticancer activity observed for ISZ-sTRAIL compared with native sTRAIL. This is the first report demonstrating that ISZ-sTRAIL expressed in *E. coli* exhibits potential antitumor activity in EML4-ALK-positive NSCLC cells, highlighting its potential as a novel therapeutic agent for this challenging patient population.

Mechanistically, TRAIL selectively induces apoptosis in malignant cells through both extrinsic and intrinsic apoptotic pathways. Specifically, TRAIL binds to death receptors DR4 and DR5, triggering the formation of the death-inducing signaling complex. This complex then recruits and activates caspase 8, thereby initiating the extrinsic apoptotic pathway. In the intrinsic pathway, activated caspase 8 further cleaves the pro-apoptotic protein Bid. The cleaved Bid subsequently promotes the activation of caspase 9, which in turn activates caspase 3, leading to apoptotic cell death [16,27]. Consistent with this canonical TRAIL-mediated apoptotic pathway, our results demonstrated that ISZ-sTRAIL treatment induced the activation of caspase 8, caspase 9, and caspase 3 and the loss of MMP in EML4-ALK-positive NSCLC cells. Moreover, the pan-caspase inhibitor Z-VAD significantly reversed ISZ-sTRAIL-induced apoptosis. This confirms that ISZ-sTRAIL exerts its antitumor effect by activating both extrinsic and intrinsic caspase-dependent apoptotic pathways in EML4-ALK-positive NSCLC cells.

The sensitivity of tumor cells to TRAIL is closely associated with the expression levels of its death receptors, DR4 and DR5 [28]. For instance, studies in nasopharyngeal carcinoma cell lines have clearly demonstrated a strong correlation between DR4/DR5 expression levels and cellular responsiveness to TRAIL [29]. Evidence has demonstrated that most locally advanced NSCLCs abundantly express DR4 and DR5, supporting these death receptors as promising therapeutic targets for NSCLC therapy [30]. However, the intrinsic expression pattern of DR4/DR5 in EML4-ALK-positive NSCLC cells remains poorly defined. In this work, we found that DR4 and DR5 were highly expressed in two EML4-ALK-positive NSCLC cell lines relative to normal lung epithelial cells. These ALK-positive NSCLC cells were also more sensitive to ISZ-sTRAIL treatment, indicating a potential correlation between DR4/DR5 expression levels and cellular sensitivity to ISZ-sTRAIL. Further work including functional studies, such as DR4/DR5 knockdown or overexpression, needs to be investigated to confirm the causal relationship. Moreover, it has been reported that DR4 expression is positively regulated by the MEK/ERK/AP-1 signaling axis [31], and relevant studies have confirmed that ALK inhibition suppresses AP-1 activity by promoting Fra-1 and c-Jun degradation, thereby downregulating DR4 expression in ALK-mutant NSCLC cells [32]. These findings suggest that ALK signaling may regulate DR4/DR5 expression and thereby modulate ISZ-sTRAIL-induced apoptosis. However, whether ISZ-sTRAIL can reciprocally regulate ALK signaling, and the potential crosstalk between these two pathways, represents an intriguing question that warrants further investigation.

Previous studies have indicated that the EML4-ALK variant 3 (V3), present in NCI-H2228 cells, may be less responsive to certain ALK inhibitors compared with variant 1 (V1) in NCI-H3122 cells, and is associated with poorer clinical outcomes and a greater propensity for drug resistance [33]. Importantly, our findings revealed that ISZ-sTRAIL exerted more potent cytotoxic activity against NCI-H2228 cells, which harbor the more aggressive V3 variant. This observation highlights the potential of ISZ-sTRAIL as a promising therapeutic strategy for ALK-positive NSCLC patients with the EML4-ALK V3 subtype, who exhibit reduced responsiveness to conventional ALK tyrosine kinase inhibitors.

In conclusion, we found that DR4 and DR5 were highly expressed in EML4-ALK-positive NSCLC cells, and ISZ-sTRAIL effectively induces apoptosis in these cells while displaying low toxicity to normal cells. Future work will further investigate its antitumor activity in vivo and efficacy against drug-resistant cells, so as to lay a solid foundation for its potential clinical application.

## 4. Materials and Methods

### 4.1. Reagents

The Bradford protein assay kit, 3-(4,5-dimethylthiazol-2-yl)-2,5-diphenyltetrazolium bromide (MTT), Trypan blue staining cell viability assay kit, Hoechst 33342, Annexin-V-FITC apoptosis detection kit, JC-1 staining kit, and Z-VAD-FMK were purchased from Beyotime Institute of Biotechnology (Shanghai, China). IPTG was obtained from Merck KGaA (Darmstadt, Germany). Primary antibodies against PARP, caspase 8, caspase 9, and cleaved caspase 3 were sourced from Cell Signaling Technology Inc. (Danvers, MA, USA). β-Actin antibody was purchased from ABclonal Technology Co., Ltd. (Wuhan, China). DR4 and DR5 antibodies were obtained from Epizyme Biomedical Technology Co., Ltd. (Shanghai, China). His tag antibody and horseradish peroxidase-conjugated goat anti-rabbit/mouse secondary antibodies were obtained from Beyotime Institute of Biotechnology (Shanghai, China).

### 4.2. Cell Culture

NCI-H2228 cells were purchased from the Cell Bank, Chinese Academy of Sciences (Shanghai, China). NCI-H3122 cells were obtained from Guangzhou Cellcook Biotechnology Co., Ltd. (Guangzhou, China), and human bronchial epithelial 16HBE cells were purchased from the Cell Resource Center of the Chinese Academy of Medical Sciences (Beijing, China). All cells were cultured in a CO_2_ incubator at 37 °C with 5% CO_2_, using RPMI 1640 medium supplemented with 10% fetal bovine serum and 1% penicillin–streptomycin solution.

### 4.3. Construction, Expression and Purification of ISZ-sTRAIL and sTRAIL Protein

The ISZ-sTRAIL recombinant plasmid contains a 6 × His-tag, a GGGGSGG linker, an isoleucine zipper (ISZ) domain (amino acids sequence: KQIEDKIEEILSKIYHIENEIARIKKLIGERE) [13], and the soluble TRAIL fragment (sTRAIL, corresponding to amino acids 114–281 of TRAIL). The ISZ-sTRAIL coding sequence was synthesized by Anhui General Biology Co., Ltd. (Chuzhou, Anhui, China) and subcloned into the pET28a(+) vector via *Nco I* and *Xho I* restriction sites. The resulting expression construct was transformed into *Escherichia coli* BL21(DE3) for recombinant protein production. Transformed bacteria were cultured at 37 °C with shaking at 220 rpm until the optical density at 600 nm (OD_600_) reached 0.6–0.8. Protein expression was induced by adding IPTG to a final concentration of 0.2 mM, followed by further incubation at 37 °C for 4 h. Cells were harvested by centrifugation, resuspended in lysis buffer (100 mM NaCl, 50 mM Tris-HCl, pH 8.0), and disrupted by sonication. The lysate was centrifuged, and the supernatant was subjected to affinity purification using Ni-NTA resin. Bound ISZ-sTRAIL protein was eluted with 500 mM imidazole and subsequently desalted. Protein concentration was determined using the Bradford assay. The purity and molecular weight of the protein were analyzed by 15% SDS-PAGE followed by Coomassie Brilliant Blue staining. The sTRAIL protein, identical to ISZ-sTRAIL except for the absence of the ISZ domain, was purified using the same procedure, with the exception that protein expression was induced with 0.6 mM IPTG for 4 h.

### 4.4. Cytotoxicity Assay

The cytotoxicity of ISZ-sTRAIL and sTRAIL was detected by MTT assay using 96-well plates. Briefly, 1 × 10^4^ cells were seeded into each well and incubated at 37 °C for 24 h. The cells were then treated with different concentrations of ISZ-sTRAIL or sTRAIL. After 48 h of incubation, 10 μL of MTT solution (5 mg/mL) was added and incubated at 37 °C for another 2 h. The supernatant was removed, 150 μL of DMSO was added, and the plate was shaken to dissolve formazan crystals. Absorbance was measured at 490 nm using a microplate reader (Multiskan FC, Thermo Fisher Scientific Inc., Waltham, MA, USA). IC_50_ values were calculated by nonlinear regression using GraphPad Prism 8.0.1 software (GraphPad Software, San Diego, CA, USA) with the log(inhibitor) vs. normalized response (variable slope) equation.

### 4.5. Trypan Blue Exclusion Assay

NCI-H3122 cells were seeded in 12-well plates at 1.5 × 10^5^ cells per well, and NCI-H2228 cells at 1.0 × 10^5^ cells per well, followed by culture for 24 h. Different concentrations of recombinant ISZ-sTRAIL were then added to each well. Subsequently, the cells were digested every 24 h up to 72 h, stained with Trypan blue, and counted.

### 4.6. Western Blot Analysis

Total proteins were separated by SDS-PAGE and electrophoretically transferred to nitrocellulose membranes. Subsequently, the membranes were blocked with 5% milk at room temperature for 1 h, followed by incubation with primary antibodies overnight at 4 °C. After washing with tris-buffered saline with 0.05% tween-20, horseradish peroxidase (HRP)-conjugated secondary antibodies were added and incubated for 2 h at room temperature. After additional washes, the membranes were developed using ECL chemiluminescence. The primary antibodies used were as follows: His antibody (1:1000, Beyotime), caspase 8 (1:1000, CST), caspase 9 (1:1000, CST), PARP (1:1000, CST), cleaved caspase 3 (1:1000, CST), DR4 (1:1000, Epizyme Biotech), DR5 (1:1000, Epizyme Biotech) and actin (1:4000, ABclonal). The original Western blot images were provided in the Supplementary Materials.

### 4.7. Hoechst 33342 Fluorescence Staining

NCI-H3122 and NCI-H2228 cells were seeded in 6-well plates at 3 × 10^5^ cells per well. The next day, the medium was replaced with fresh medium containing different concentrations of recombinant ISZ-sTRAIL. After 48 h (24 h for NCI-H2228), cells were washed three times with PBS, fixed with 4% paraformaldehyde, and stained with Hoechst 33342. Nuclear changes in cells were observed under an inverse fluorescence microscope (Micro-shot Technology Co., Ltd., Guangzhou, China) at 40× magnification.

### 4.8. Flow Cytometry Assay

NCI-H3122 and NCI-H2228 cells were seeded in 6-well plates at a density of 1.5 × 10^5^ cells/mL and cultured overnight. The cells were then treated with various concentrations of ISZ-sTRAIL for 24 h or 48 h. Both adherent and detached cells were collected, washed with PBS, and stained with Annexin V-FITC and propidium iodide (PI) for 20 min at room temperature. Apoptosis was evaluated by flow cytometry using a CytoFLEX flow cytometer (Beckman Coulter Inc., Brea, CA, USA).

### 4.9. JC-1 Staining

JC-1 staining was performed according to the manufacturer’s protocol. Briefly, after ISZ-sTRAIL treatment for 24 h, cells were incubated with JC-1 staining solution for 20–30 min at 37 °C, washed twice, and then visualized under a fluorescence microscope. CCCP (10 µM) was used as a positive control for MMP depolarization.

### 4.10. Statistical Analysis

All quantitative data were repeated at least three times. Data were presented as mean ± standard deviation (SD) (unless otherwise stated) and analyzed by GraphPad Prism 8.0.1 software (GraphPad Software, San Diego, CA, USA). Statistical significance between the experimental and control groups was assessed by one-way analysis of variance (ANOVA), and comparisons between two groups were performed using two-tailed Student’s *t*-test. *p* < 0.05 was considered statistically significant.

## Figures and Tables

**Table 1:** IC_50_ values of ISZ-sTRAIL and sTRAIL against different cell lines.

Protein	Cells (IC_50_, nM)		
	16HBE	NCI-H3122	NCI-H2228
ISZ-sTRAIL	>1000	14.98 ± 3.34	4.51 ± 0.22
sTRAIL	N.D.	53.27 ± 6.38	22.37 ± 1.34

N.D., not detected; IC_50_ values are presented as mean ± SEM from at least three independent experiments, and the dose–response curves were fitted by nonlinear regression.

## Data Availability

All data are available within the manuscript and upon request to the corresponding authors.

## Supplementary Materials

The following supporting information can be downloaded at: https://www.mdpi.com/article/10.3390/molecules31111870/s1.
