# Supplementary material for: Identification of ISZ-sTRAIL Protein as a Potent Anticancer Agent for EML4-ALK-Positive Non-Small-Cell Lung Cancer
Source: Molecules. 2026 May 29;31(11):1870. doi: 10.3390/molecules31111870 (PMC13257729; doi:10.3390/molecules31111870)
Supplement: Supplementary file 1 [file molecules-31-01870-s001.zip › molecules-4268550-supplementary.pdf]

Identification of ISZ-sTRAIL Protein as a Potent Anticancer Agent for EML4-ALK-Positive Non-Small-Cell Lung Cancer

Junfeng Hu, Junhui Guo, Tian Qin, Xiuping Mao, Zi Liu* and Liang Ma *

Suplementary Material

Original Western blots images.

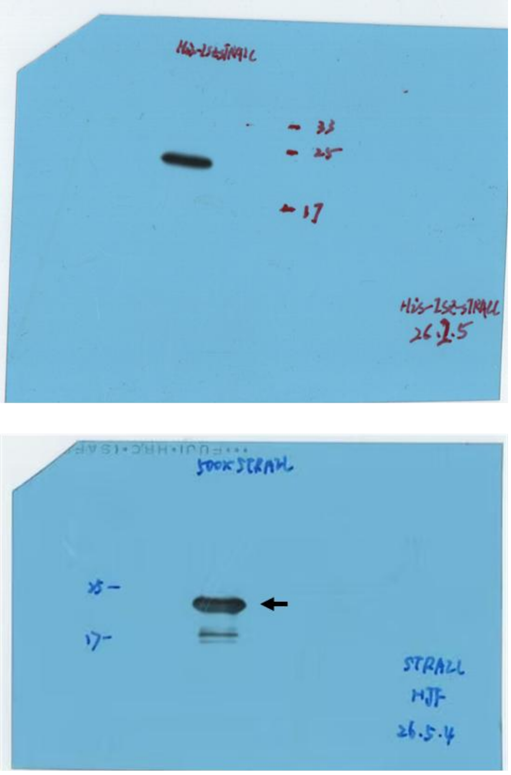

Figure 1d

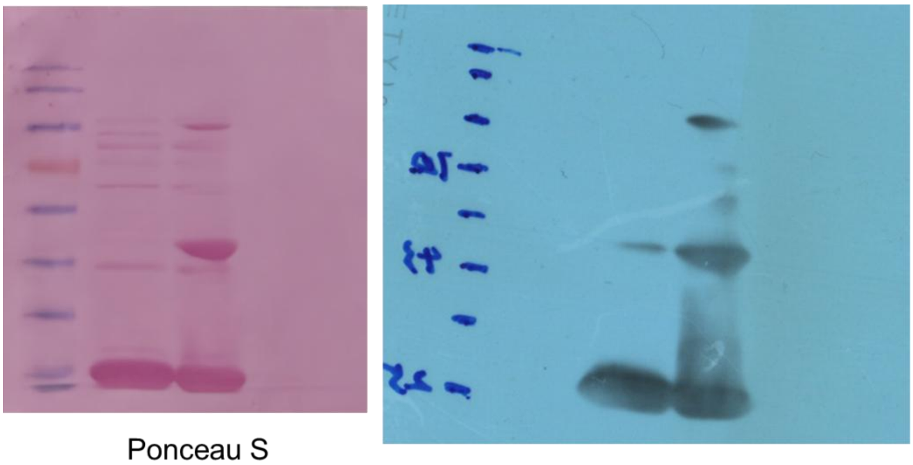

Ponceau S

Figure 1f

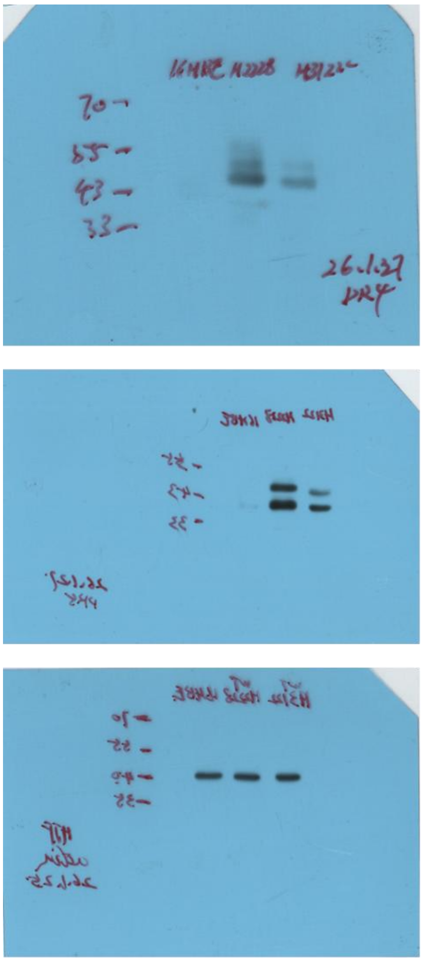

DR4 (40-55 kD)

DR5 (40 kD, 49 kD)

Actin (42 kD)

Figure 2a

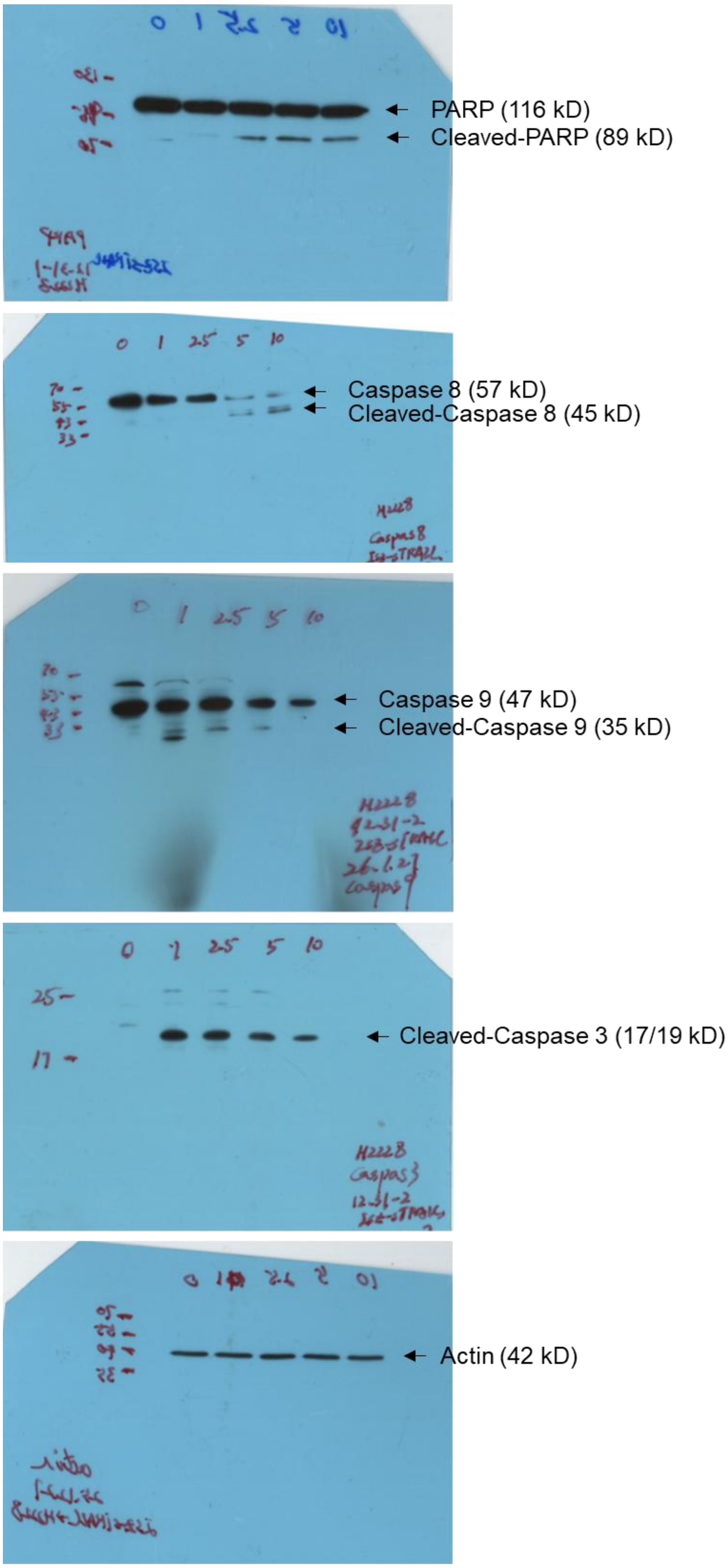
PARP (116 kD)
Cleaved-PARP (89 kD)
Caspase 8 (57 kD)
Cleaved-Caspase 8 (45 kD)
Caspase 9 (47 kD)
Cleaved-Caspase 9 (35 kD)
Cleaved-Caspase 3 (17/19 kD)
Actin (42 kD)

Figure 5a

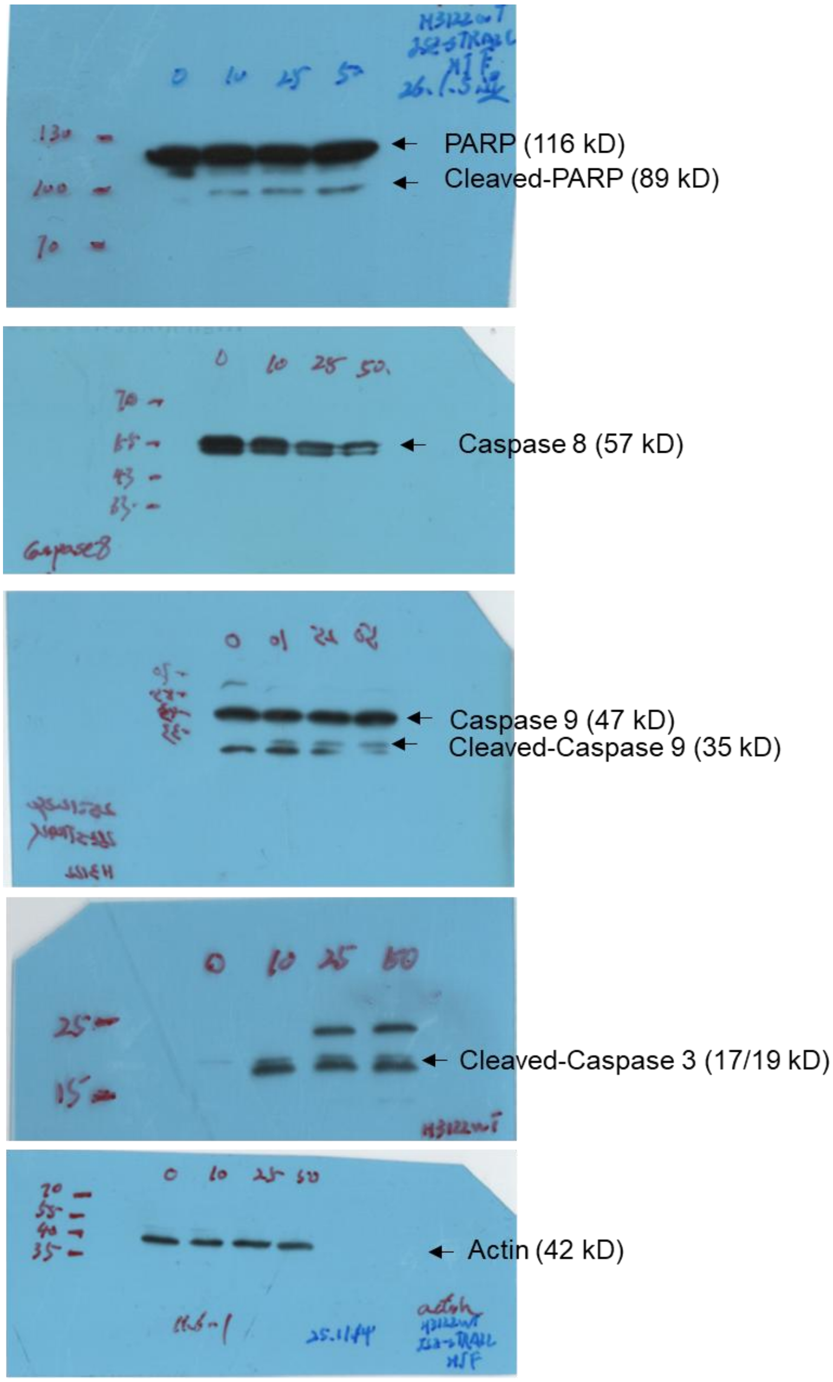
PARP (116 kD)
Cleaved-PARP (89 kD)
Caspase 8 (57 kD)
Caspase 9 (47 kD)
Cleaved-Caspase 9 (35 kD)
Cleaved-Caspase 3 (17/19 kD)
Actin (42 kD)

Figure 5b

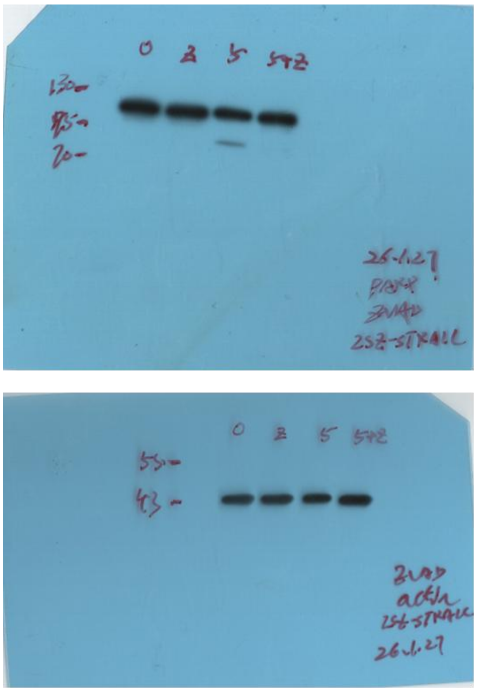

Figure 6c

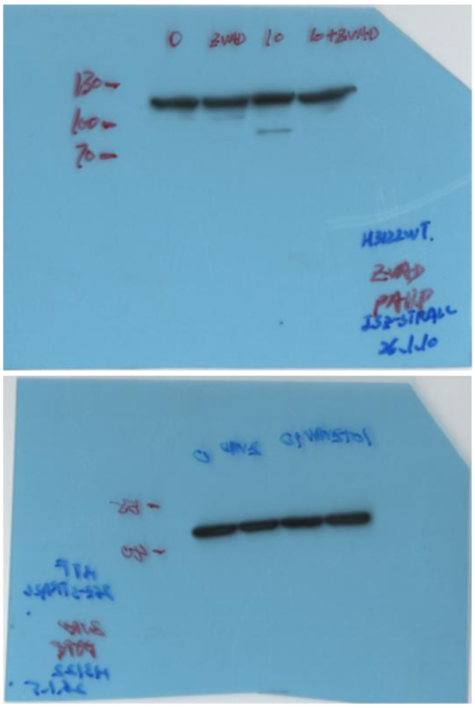

Figure 6d
